# Editorial: Hereditary and acquired disorders of calcium homeostasis

**DOI:** 10.3389/fendo.2022.1124762

**Published:** 2022-12-29

**Authors:** Sami A. Sanjad, Hala Tfayli, Bilal Aoun

**Affiliations:** ^1^ Department of Pediatrics, Division of Nephrology, American University of Beirut, Beirut, Lebanon; ^2^ Department of Pediatrics, Division of Endocrinology, American University of Beirut, Beirut, Lebanon

**Keywords:** hypocalcemia, hypercalcemia, parathyroid hormone, vitamin D, rickets, disorders of calcium homeostasis

Several players interact to maintain normocalcemia; 1) Parathyroid hormone (PTH) causes calcium resorption from bones, 2) Active vitamin D (calcitriol) facilitates calcium absorption from the small intestine, 3) Calcitonin plays a minor role in calcium metabolism by its anti PTH effect and 4) FGF23 inhibits phosphate re-uptake and the expression of 1-alphahydroxylase (CYP27B1) in the renal proximal tubules (1).

Hypocalcemia and hypercalcemia usually occurring from imbalances in the above mechanisms, present challenging diagnostic, and therapeutic issues to the pediatrician. Both acute and chronic, syndromic, and non-syndromic, hereditary, and acquired forms, are recognized.

Hypocalcemia may occur in the neonatal period, both early and late, and the mechanisms involved are only partially understood. Hypocalcemia in infants and older children is usually related to hypoparathyroidism or inactive PTH, severe vitamin D deficiency or inactivating mutations in the vitamin D nuclear receptor. A rare cause is due to activating mutations in calcium sensing receptor (CaSR) which leads to autosomal dominant familial hypercalciuric hypocalcemia (2, 3).

Hypercalcemia may be due to increased intestinal absorption (vitamin D excess, granulomatous diseases), increased bone resorption (hyperparathyroidism, acidosis, malignancies, immobilization), William’s syndrome, thyroid disease and pheochromocytoma (4).

The current issue of Frontiers in Endocrinology is dedicated to disorders of calcium metabolism leading to hypocalcemia and hypercalcemia.

Five articles, including two case reports, address various conditions associated with hypocalcemia (4 articles) and hypercalcemia (1 article). Al Homyani and Alhemaiani present the case of a 4-year-old boy from Saudi Arabia with severe rachitic bone disease and hypocalcemia due to vitamin D dependent rickets type 1A (VDR1A). Genetic testing revealed a novel homozygous missense mutation (R459C) in the CYP27B1gene. The patient had a good biochemical response to alfacalcidol with normalization of his serum calcium, phosphate, and alkaline phosphatase. The same mutation has been reported previously in a Chinese boy but in a compound heterozygote form (R459C, G73W) (5). Fortin et al. provide convincing evidence of the benefits of neonatal screening for VDR1A in an inbred population in the Saguenay-Lac-Saint-Jean region in Quebec, Canada. Thus, in a cohort of 2000 newborns tested for VDR1A, they found a carrier rate of 1/29 for the c.262delG in the CYP27B1 gene which causes VDR1A, suggestive of a founder effect. One baby was found to be homozygous, which allowed for early initiation of treatment and prevent the harmful consequences of metabolic bone disease. Since only this pathogenic variant has been detected in this population with a relatively high prevalence, genetic screening for this variant was deemed safe feasible and cost effective. Calcium is an important ion in neonatal nerve conduction. Neonatal hypocalcemia might be either symptomatic leading to jitteriness, and even seizures in some cases, or asymptomatic detected on simple blood sample. In both instances, identifying and treating hypocalcemia is of great importance to avoid serious complications. Identifying the cause of hypocalcemia by performing genetic studies is often necessary to rule out syndromic causes (6). In a review article, Huang et al. evaluated 1029 patients admitted to the neonatal intensive care unit. Sixteen babies developed neonatal hypocalcemic seizures. They classified their type of seizures as syndromic and non-syndromic according to the etiologic findings. Moreover, 5 out of the 16 patients had underlying syndromic features, three had DiGeorge syndrome, one Kleefstra syndrome, and one Alstrom syndrome. The remaining 11 were non-syndromic and were mostly associated with vitamin D deficiency. Unsurprisingly, the authors concluded that newborn babies with hypocalcemic seizures associated with syndromic entities had worse neurodevelopmental outcomes and were more refractory to therapy, highlighting the importance of early identification of such cases by performing genetic studies for diagnosis and to start early treatment.

Pseudohypoparathyroidism and related disorders belong to a group of rare diseases sharing an impaired downstream signaling of Gsα-protein-coupled receptors. Affected patients present with various resistance to PTH and/or to other hormones, ectopic ossifications, brachydactyly type E, early onset obesity, short stature and cognitive difficulties. The term inactivating PTH/PTHrP signaling disorders (iPPSD) has been proposed by Thiele et al. as a novel nomenclature for these various disorders (7). Demaret et al. described the case of a 10- month-old male infant of Turkish origin born to consanguineous parents, presenting with neurodevelopmental delay and seizures. The patient had several syndromic features, and the diagnosis of PTH resistance was made on the basis of severe hypocalcemia, hyperphosphatemia, elevated PTH and normal vitamin D levels. On follow up the patient developed Arnold-Chiari type malformation requiring a ventriculoperitoneal shunt. Whole exome sequencing revealed a novel homozygous, likely pathogenic, missense mutation p. (Asp 241Glu) in the PTH1R gene. Parents were found to be heterozygous for the same mutation. This case diagnosed for the first time PTH resistance in a child with biallelic PTH1R mutation thereby extending the clinical spectrum of inactivating PTH/PTHrP signaling disorders. It also raises the question if other types of mutations might lead to additional phenotypes.

Primary hyperparathyroidism (PHPT) is rare in children with a reported incidence of 2-5 per 100,000 compared to 1 per 1000 in adults (8). While most adults with PHPT are asymptomatic, children have a more severe presentation and greater morbidity including failure to thrive, skeletal abnormalities, nephrolithiasis, nephrocalcinosis and abdominal and bone pain (8, 9). Cinacalcet, a type II calcimimetic that acts as an allosteric modifier reducing the threshold for CaSR activation by calcium, is licensed in Europe for use in dialysis patients with secondary hyperparathyroidism above the age of 3 years (10). Its use in PHPT in children has been limited to off-label single case and small series reports, and data on its long-term use is scarce. Bernador et al. describe the French experience in off-label use of cinacalcet in 18 children with PHPT (10 with inactivating mutations in *CaSR*). The age range was 2-14 years at initiation of treatment, and the duration of follow-up reached up to 4.3 years. In this series cinacalcet use was effective in most patients, and side effects limited to nephrolithiasis in one patient. While this is a promising report including a relatively large number of young patients with a rare condition, there is variability in the doses used, the age at initiation of treatment and the duration of follow-up. This report opens the door for developing unified protocols that may be used in larger multinational studies.

## Author contributions

All authors listed have made a substantial, direct, and intellectual contribution to the work and approved it for publication.
